# An Injury Prevention Perspective on the Childhood Obesity Epidemic

**Published:** 2009-06-15

**Authors:** Keshia M. Pollack

**Affiliations:** Department of Health Policy and Management, Center for Injury Research and Policy, Johns Hopkins University Bloomberg School of Public Health

## Introduction

Physical activity during childhood and adolescence is associated with many benefits, including decreased adiposity, improved cardiovascular health and fitness, reduced symptoms of depression and anxiety, greater global self-concept and esteem, and improved academic performance (1,2). Physical activity is also an essential component of a multifaceted approach to prevent or control obesity in children.

Many studies have investigated barriers and facilitators of physical activity in children, but few mention injury prevention and control. Injury prevention is characterized by the use of multidisciplinary approaches (eg, epidemiology, policy, behavioral science) to create safer products and environments. Surprisingly, as interventions are developed to increase physical activity among children by promoting the use of playgrounds, bicycle use, walking, and participation in sports and recreation, information about preventing injuries during these activities is scarce. This lack of attention to injury prevention is a missed opportunity for advocates of physical activity to address parents' concerns about safety, which may be a barrier.

The purpose of this paper is to initiate discourse on the value of including injury prevention and control as part of public health strategies to increase physical activity among youth. This article describes the connection between injury prevention and physical activity, proposes the benefits of using an injury prevention framework when developing physical activity interventions, and recommends that an injury prevention perspective on the childhood obesity epidemic be used to guide future research.

## Connecting Injury Prevention and Physical Activity

Unintentional injuries are the leading cause of death for children aged 19 years and younger. Most deaths result from motor vehicle crashes, falls, burns, drowning, and poisonings (3). The economic impact of childhood injuries is substantial. Injuries to children result in an estimated $14 billion in lifetime medical spending, $1 billion in other resource costs (eg, caring for injured children), and $66 billion in present and future work losses (4).

Though some may not recognize it, the association between injury prevention and physical activity has existed for years. Several injury prevention interventions have modified the built environment, making it easier for people to be active safely. Traffic-calming measures, such as sidewalks, have enhanced pedestrian safety. Laws that restrict vehicle speeds encourage safe biking and walking. Regulations, such as bans on some forms of tackling, have decreased catastrophic sports injuries. Although the effectiveness of these interventions relies on adequate enforcement, they create safe places and opportunities for people to be active.

Advocates of physical activity to prevent or control obesity often overlook the importance of injury prevention when developing interventions. This is alarming because injuries are a reason that people stop participating in physical activity (5). Decreasing or eliminating the risk of injury may encourage people to initiate physical activity or continue being physically active.

One of the most compelling examples of the connection between injury prevention and physical activity is playground safety. Some of the earliest playgrounds were filled with hazardous equipment that resulted in many serious fall-related injuries to children. During the 1970s the Consumer Product Safety Commission first alerted the public that playground design can contribute to fractures, lacerations, and abrasions of children seen in the emergency department (6). The response to this public health problem was to modify the environment. Today, most playgrounds are constructed with impact-absorbing surfaces, and slides are at or below a maximum height; overall, they are now safe places for children to play and be active.

Imagine if the risk of playground-related injury were high at a particular location. Parents likely would not allow their children to play there, especially because safety is a factor for parents in selecting places for their children to play (7). Knowing that safety is a barrier to physical activity is critical because efforts to increase use of a playground will be unsuccessful unless actual and perceived injury risks are recognized. The nexus is clear: reduce the risk of playground-related injury, increase actual and perceived safety, and promote playgrounds as a place for children to be active. Injury prevention specialists have figured out that playgrounds can be designed and maintained to markedly reduce the risk of injury, and those who promote use of playgrounds as a way to increase youth physical activity can benefit from these lessons.

Another example of the connection between injury prevention and physical activity is the Safe Routes to School (SRTS) programs. These programs are designed to enable and encourage children's physical activity through safe walking or bicycling to school (8). Federal funds are available for programs and projects that change the environment to support safe commuting to schools. Traffic signals, crossing guards, and pedestrian overpasses are just a few of the interventions that are part of the SRTS programs. These interventions are also often part of community pedestrian injury prevention programs.

Injury prevention is also relevant when considering the social environment, which may affect the likelihood of a child actively commuting to school. Intentional injuries, such as violence and crime, may affect activity levels because of perceived fear. In fact, parents often cite both traffic safety concerns and crime as predominant reasons for their children not walking to school and participating in outdoor activity (9). Before implementing SRTS programs as a means to increase youth physical activity, unintentional and intentional injury risks along school routes need to be identified and reduced.

## An Injury Prevention Framework Applied to Physical Activity

William Haddon Jr, one of the founders of injury epidemiology, developed Haddon's 10 Basic Strategies (countermeasures) for Injury Prevention (10). Haddon's countermeasures help us understand injuries and identify multiple countermeasures to prevent them, and they provide public health professionals with a framework to identify risk factors or devise preventive strategies. The strategies range from preventing the hazard to providing rehabilitative services for the injured person.

Advocates who develop physical activity interventions that target childhood obesity can glean key elements from these countermeasures. During the planning stage, program planners can consult Haddon's strategies to ensure they are included in their interventions to increase physical activity. Because the risk of pedestrian injury is a barrier for parents in letting their children actively commute to school, program developers of the SRTS programs, for example, can refer to Haddon's framework to consider multiple strategies for reducing the risk of pedestrian injury. In another example, efforts that encourage children to walk or bicycle to school could benefit from Haddon's strategies by thinking about how

- Traffic-calming devices can be installed to reduce the speed of vehicles.
- Walkways and pedestrian overpasses can be built to separate cars from pedestrians and bicycles.
- Helmets could be required and children and parents educated about their use and fit.

These strategies may seem obvious. However, the benefit of using this framework is that it systematically helps public health professionals to develop programs while preventing the risk of injury. Furthermore, these strategies also suggest the many ways that the risk and severity of injuries, and their resulting conditions, can be mitigated.

## Recommendations for Future Research

Several factors affect child physical activity, but less is known about how injury is related to physical activity and the need for new knowledge is evident. Professionals from the fields of childhood obesity and injury prevention should be working together to explore injury risks during physical activity and to understand safety concerns of both parents and children. A few actions would strengthen the research base in this area:

1. *Document childhood injuries during physical activity.* More studies are needed that quantify the risk, prevalence, and incidence of injuries related to physical activity among children. A US Department of Health and Human Services report on physical activity guidelines listed areas for future research, including the lack of understanding about the risk for unintentional injuries for both active and inactive people (11). A better understanding is also needed of how fear of injury and perceived safety, by parents and children, may be a deterrent for youth physical activity. Recently published studies have begun to explore these areas (12-15). However, research questions related to sports, recreation, and exercise-related injuries remain (16).

2. *Investigate the risk and distribution of injury in studies of access and opportunities for places for youth to engage in physical activity. *Research that highlights access and availability of child recreational and free-play facilities (eg, parks, playgrounds, basketball courts) should explore issues not only related to proximity but also to injury. Studies that identify the mechanism, etiology, and burden of injury should be part of the literature on access and availability of opportunities for child physical activity. Moreover, violence, crime, and intentional injury as barriers to physical activity warrant further exploration.

3. *Explore the implications of obesity for safety equipment fit and availability.* Studies are needed that explore the effect of obesity on use, fit, and availability of safety equipment. One of Haddon's prevention strategies is to separate people from the hazard by interposing a material barrier. Children wear protective gear (eg, bicycle helmets, kneepads) during participation in many sports. The availability and cost of safety equipment in larger sizes and problems with acceptability, comfort, and fit for overweight and obese children are topics that warrant more research.

## Conclusion

Physical activity has many benefits for children. However, merely encouraging parents and children to be active, without first measuring the risk for injury and implementing strategies to reduce those risks, is not enough. Ensuring that the hazards children encounter while being physically active are decreased and their risk for injury is reduced is the responsibility of the public health community.

Injury prevention and childhood obesity share a common goal of improving children's health, and public health programs to improve child health should be coordinated, rather than working at cross purposes. Multidisciplinary interventions that share the goal of keeping children safe and healthy could pave the way for new partnerships, stretch scarce public health resources, and tackle these serious public health threats facing our youth today.
